# The Physiological Anatomy and Physiology of Man. Part the Third

**Published:** 1847-10-01

**Authors:** 


The Physiological Anatomy and Physiology of Man. Part the Third. By *Robert Bentley Todd,* M.D., F.R.S., Professor of Physiology, and *William Bowman,* F.R.S., Demonstrator of Anatomy in King's College, London. John W. Parker, 1847.

We are happy to announce the appearance of the first portion of the second and concluding volume of this important work; and trust that the remaining part will, as the advertisement states, be speedily published. The part before us contains a very interesting account of the organs of smell, vision and hearing, including their structural anatomy and physiology; the anatomy and functions of the several encephalic nerves of the great sympathetic, are next given; and, lastly, the ogans of digestion are partly investigated. We propose to lay before our readers a concise account of the many new facts connected with the Organs of Sense, with which the authors have enriched their pages; but to accomplish this object, it will be necessary to refer to the latter part of the first volume, containing the Organs of Touch and Taste, published about two years since, and which we have not hitherto had an opportunity of noticing. We must, however, in the first place congratulate Dr. Todd and Mr. Bowman, and especially the latter gentleman, that they have been able, by their admirable and skilful observations, to throw so much new light on the delicate organization of many important parts; a merit this of no ordinary kind, when it is recollected that the intimate texture of the organs to which we refer, has been made the subject of laborious and repeated investigation by several of the most distinguished microscopists of Europe.

Without entering into any metaphysical speculations, we may briefly consider, as constituting the most fitting introduction to the subject matter of the work before us, the nature of sensation in general, and especially the relations existing between the mind and the external objects of sense; and we are the more strongly prompted to this, by the consideration that some of the errors of former times still linger among us; and especially because, although the mechanism of perception, as we may for the moment be permitted to term it, is tolerably well understood by physiologists, it is but very imperfectly apprehended by persons in general, even in many instances by those who have paid some attention to the subject. The authors preface their account of the special senses by the following definition :—

"Sensation is an affection of the mind occasioned by an impression made on certain parts of the nervous system, hence called sensitive. A state of the sensitive organs, and a corresponding perception by the mind, must concur to produce sensation: either condition may exist alone, but then the phenomenon is not a true sensation, in the acceptation here given to the word. Thus, light falling on the eye in sleep excites the whole visual sensitive apparatus, while the organ of perception is inactive: on the other hand, in dreams, vivid pictures of objects float before the mind, and are referred by it to the external organ, which may be all the while entirely quiescent." Vol. I, p. 402.

Sensation is thus properly regarded as a phenomenon of *consciousness*, an acceptation of the term which definitively excludes what has been called, after Bichat, organic sensibility. But, although the authors are correct as far as they have gone, they have not explained what it is which, in the act of perception, is presented to the mind; an explanation which is the more demanded, since, in a passage immediately following, it is noticed as a remarkable circumstance that "the organ of the mind itself does not appear capable of undergoing sensory excitement;" or, in other words, of receiving directly impressions from the external objects of sense. The fact is that, in every kind and form of sensation, the mind does not take cognizance of the properties of external objects, but of the altered state of the nerve of sense induced by the impression made upon it, as by light, sound, &c. This truth, which is essential to a just appreciation of the whole matter, is thus clearly stated by Professor Müller in his Physiology: "sensation consists in the sensorium receiving, through the medium of the nerves and as the result of the action of an external cause, a knowledge of certain qualities or conditions, not of external bodies, *but of the nerves of sense themselves*." In order then to excite the act of perception, the impression must in every instance be made on a nerve of sense; and this being so, the wonder ceases as to the cerebral hemispheres, the seat of all perception, being themselves incapable of perceiving impressions made directly upon them by external bodies. But we learn, moreover, from this that, although many persons conceive that what are called "subjective" and "objective" sensations are essentially distinct modes of sensation, in one case the mind taking cognizance of a condition of the nerve, and in the other of the quality of the external object, they in reality only differ from each other as to the manner in which the nerve is excited; in subjective sensation, a stimulus of an unusual kind is applied, as when the retina is pressed upon by the finger applied to the globe of the eye, or when the auditory nerve is excited by some deranged state of the circulation inducing tinnitus aurium; whereas, in objective sensation, what is considered the natural stimulus is applied.

In describing the organ of taste, the authors have given a most interesting account of the various forms of papillæ placed on the tongue. The most remarkable fact is, that the conical or filiform papillæ, distinguished on the dorsum by their whitish colour, are not only provided with an abundant epithelium frequently composing two thirds of their length, but actually present, at least a few of them, minute hairs, the largest of which are $\frac{1}{10}$ of an inch long, and from $\frac{1}{2000}$ to $\frac{1}{3000}$ of an inch thick. There can be little doubt that these papillæ are subservient rather to touch than to taste; and they may, by their structure and partial mobility, fulfil the office assigned to them by the authors, that, namely, of directing the muscular actions of the tongue during mastication, and thus promoting the almost manual dexterity of the organ in dealing with minute particles of food.

The chapter treating on the Minute Anatomy of the Eye, contains several new facts, which must prove most interesting as regards both the functions and diseases of the various component parts of this most complex organ. In the first place, as respects the cornea, it may not be superfluous to state, although the greater part of this has been for some time known, that it is composed of five coats or layers, clearly distinguishable from each other; these are the conjunctival layer of epithelium, the anterior elastic lamina, the cornea proper, the posterior elastic lamina, and the epithelium of the aqueous humour, or posterior epithelium. The conjunctival epithelium may always be obtained from a fresh eye by gently scraping the surface of the cornea; it consists of three or four layers of superposed particles, inclining to the columnar form where they rest on the anterior elastic lamina. "It is in this epithelium that particles driven with force against the eye generally lodge, and it is easily detached by the instrument used to extract them. Vessels shooting into the cornea in disease* lie under it, and small ulcers are formed by its destruction."—Vol. II., p. 20.

The cornea proper, or lamellated cornea, consists of a peculiar modification of the white fibrous tissue, which is continuous with that of the sclerotic. The lamellæ, sixty and upwards in number, formed of these fibres, are intimately united to one another by processes of a similar texture, which produce, however, a very delicate, tubular structure, now for the first time thus described.

"The resulting areolæ, which in the sclerotic are irregular, and on all sides open, are converted in the cornea into tubular spaces, which have a very singular arrangement, hitherto undescribed. They lie in superposed planes, the contiguous ones of the same plane being for the most part parallel, but crossing those of the neighbouring planes at an angle, and seldom communicating with them. The arrangement and size of these tubes can be shown by driving mercury, or coloured size, or air, into a small puncture made in the cornea. They may also be shown under a high power by moistening a thin section of a dried cornea, and opening it out by needles. The tissue forming the parietes of these tubes is membranous rather than fibrous, though with the best glasses a fibrous striation may be frequently seen, both in the laminæ separating the different series of tubes, and in that dividing those of the same layer from each other. By acetic

---

* In health it is now known that the cornea contains no blood-vessels.

acid, also, the structure swells, and displays corpuscles resembling those apparent in the white fibrous tissue. Such is the lamellar structure of the cornea, which makes it so much easier to thrust an instrument horizontally than vertically into its substance. The tubes or elongated spaces of which we have spoken, are not distended with any fluid, but are merely moistened in the same way as the areolæ of ordinary areolar tissue. A perfectly fresh and transparent cornea is rendered opaque by pressure, but it regains its brilliance on the removal of the compressing force. Some have supposed this to result from the expulsion of fluid from between its laminæ; but that the opacity is owing simply to a derangement of the elementary parts of its structure is plain from the fact, that the same phenomena are exhibited by a section however thin, immersed in water, and deranged by stretching." P. 20.

The authors regard the iris as a process of the choroid, with which it is continuous, although the structure is of course much modified. The connexion of this imperfect diaphragm with the cornea is known to be very firm, but the minute texture uniting them together has not heretofore been investigated. The anterior surface of the iris becomes continuous in the following manner with the posterior elastic lamina of the cornea.

"This lamina near its border begins to send off from its anterior surface, or that towards the laminated cornea, a network of elastic fibres, which stretch towards the border, becoming thicker as they advance, until at length the entire thickness of the lamina is expended by being converted into them. These fibres then bend backwards from the whole circumference of the cornea, to the circumference of the front of the iris, and are there implanted, passing in this course across the rim of the anterior chamber, and through the aqueous humor. They are seen more easily in some animals than in others, forming a regular series of pillars around the anterior chamber. Behind these there is a more diffused union of the tissue of the iris with the sclerotic, by means of the ciliary ligament." P. 25.

As regards that most interesting point—the muscularity of the iris, it is found, when microscopically examined, that its principal tissue so nearly resembles in its anatomical characters the unstriped muscular fibre, that it may be considered as a variety of that tissue.

"The principal direction taken by the fibres is towards the pupil, although they are more or less meandering and interlacing in this course. Arrived near the pupil, they appear to join, and form indistinct arches. In many instances it is easy to detect a set of circular fibres, either gathered into a principal bundle near the pupil or more diffused, but always lying in front of the others. These seem to answer to the circular fibres of the bird's iris, which are of the striped variety, and occupy the front of the membrane. There may also be usually distinguished in the very thin margin of the pupil an arrangement of fibres more circular than radiating." P. 26.

Placed behind the ciliary ligament and covering the outside of the ciliary body, is a very interesting structure, of a grayish and transparent appearance, which was described by many of the older anatomists, and especially by that admirable writer Porterfield, as muscular, a view adopted by Wagner, and confirmed in the present work by direct microscopic examination.

"It belongs to the unstriped variety of muscle, and its fibres appear to radiate backwards from the junction of the sclerotic and cornea, and to lose themselves on the outer surface of the ciliary body. The more superficial fibres are in contact with, but scarcely adhere to, the sclerotic, and are inserted into the posterior part of the ciliary body; while the deeper ones seem to dip behind the iris to the more prominent parts of the ciliary processes which approach the lens. The ciliary muscle must have the effect of advancing the ciliary processes, and with them the lens, towards the cornea." P. 27.

The authors justly observe that the muscular nature of this *ciliary muscle,* as it is called, is confirmed by its anatomy in birds, where it is largely developed, as noticed by Sir P. Crampton. In this class its fibres, we are informed, are like those of the iris in the same animals of the striped variety, and are supplied by ciliary nerves traversing the muscle in a circular direction.

A very beautiful representation is given of the complex organization of the retina, to which we would call the particular notice of our readers. This delicate nervous expansion has of late years been very minutely described, and various layers of gray and fibrous matter have been discovered. According to the authority before us, the following parts are to be detected proceeding *from within, outwards:* 1. A layer of transparent cells, connecting the hyaloid tunic and retina; 2. A gray nervous layer, composed of (*a*), a fibrous lamina, and (*b*), a vesicular lamina; 3. A *granular layer,* divisible by great care into three laminæ; 4. The membrana Jacobi. The great importance of this, the essential part of the eye, induces us to extract the following description of the constituent parts just enumerated.

" The first part of the retina to be described is the *fibrous gray layer,* which forms the immediate continuation of the optic nerve, and which is seated on its inner surface. This is a layer of fibrous character, radiating from the end of the optic nerve, and apparently consisting of the tubular fibres of that nerve deprived of their white substance; that is, being no longer tubular and white, but solid and gray, and united together more or less into a membrane. This at least seems to us to be certain, that the white substance of Schwann does not exist in the nervous substance of the retina, but ceases as the nerve perforates the sclerotic. It has been particularly described as existing in the retina of the rabbit; but the fact seems to be, that in this animal the nerve does not end in the retina till some way within the globe, for, after bifurcating and spreading out as a white streak within the choroid, the bundles of nerve-tubes suddenly lose their white lustre, and assume the appearance of the gray fibres of the layer now under consideration. These bundles, both in animals and man, may be seen to anastomose in a close plexiform manner, especially near the optic nerve, and finally constitute a thin sheet, which becomes thinner and less fibrous as we trace it forwards, until at length it can be no longer discerned. This fibrous gray layer of the retina is united to the hyaloid membrane, containing the vitreous humor, by a layer of nucleated cells almost perfectly transparent, and sometimes very difficult of discovery on that account. It is to be remarked, that the fibrous gray layer is the only nervous element of the retina existing over the extremity of the optic nerve where it enters the globe—a spot incapable of vision. Immediately around this spot, the other layers commence which have now to be described, and the first of these is the *vesicular gray layer.* This layer is on the outer surface of the fibrous layer, and so intimately blended with it, that it might almost seem as if the fibres successively terminated in it. The vesicular layer is thicker behind, and gradually thinner forwards. It very accurately corresponds with the vesicular matter of the convolutions of the cerebrum, consisting of a finely granular matrix with interspersed very delicate vesicles, furnished with pellucid globular nuclei of characteristic appearance.

" The blood-vessels of the retina, which are thickly distributed, belong solely to the fibrous and vesicular layers now mentioned. The central artery of the retina, after entering the globe in the axis of the optic nerve, sends four or five radiating branches, which almost immediately perforate the fibrous layer and spread out in a beautifully arborescent manner, as a capillary network in the substance of the vesicular stratum. After slight maceration, it is easy to wash the nervous material out of the meshes of the vessels; and they then form a vascular layer, but which it is hardly correct to describe as a distinct lamina of the retina. They are merely the nutrient vessels of the part, and are the representative of the close network of the gray substance of the cerebral convolutions. Their wall is a diaphanous membrane with nuclei projecting at intervals, and the meshes average $\frac{1}{400}$ of an inch diameter.

"Behind the vesicular gray layer is the *granular layer*, a term we shall apply to it, because it seems to consist of a close aggregation of small granules, which refract the light more powerfully than the neighbouring parts, and have scarcely any appearance of intervening matrix; they might be regarded perhaps as analogous to the nuclei of cells, and much resemble a layer of granules in the substance of some of the cerebral convolutions, and of the laminæ of the cerebellum. They are made more evident by acetic acid. This layer is divided into two, of which the inner is much the narrower, by a *pale stratum*, which can only be seen by very careful manipulation." P. 30.

Another subject of interest relates to the real nature of the yellow spot or central foramen of Sœmmerring. It only exists in man and the monkey among mammalia, though an analogous part has been found by Dr. Knox in reptiles. It is thus described: this spot consists of a slight projection of the retina, having a minute aperture in the summit, through which the interior of the globe can be seen, when the sclerotic and choroid have been cautiously removed; as to structure, it appears that the fibrous lamina of the optic nerve cannot be traced quite up to the spot, whilst the nucleated cells of the fibrous plexus already described, closely set together, cover its whole surface. This absence of the fibrous element, in a part so exquisitely sensitive as the foramen of Sœmmerring, seems to indicate that, in the operation of the senses, the first and essential impression is made on the gray element of the nervous system; or, in other words, that the peripheral as well as central part of the system is provided with vesicular matter, the nerve-fibres being merely interposed to transmit the impression.

In the chapter relating to the Organ of Hearing, an elaborate account is given of the minute structure of the cochlea, which, to be clearly understood, requires the assistance of the admirable wood-cuts by which the text is illustrated. After describing a peculiar arrangement connected with the osseous portion of the lamina spiralis, and which it is proposed to call the *denticulate lamina*, the authors proceed to notice a semi-transparent structure attached to the membranous zone of the cochlear septum, and which, presenting a muscular texture, is termed the *cochlearis muscle*.

"At its outer or convex margin, the membranous zone is connected to the outer wall by a semi-transparent structure. This gelatinous-looking tissue was observed by Breschet, and is indeed very obvious on opening the cochlea; but we are not aware of any one having hinted at what we regard to be its real nature. The outer wall of the cochlear canal presents a groove, ascending the entire coil, opposite the osseous zone of the lamina spiralis, and formed principally by a rim of bone, which, in section, looks like a spur, projecting from the tympanic margin of the groove, the opposite margin being very slightly or not at all marked. This groove diminishes in size towards the apex of the cochlea. It gives attachment to the structure in question, by means of a firm dense film of tissue, having a fibrous character, and the fibres of which run lengthwise in the groove, and are intimately united to it, especially along the projecting rim. From this *cochlear ligament,* the cochlearis muscle passes to the margin of the membranous zone, filling the groove, and projecting into the canal, so as to assist in dividing the tympanic and vestibular scalæ from one another, and thus forming in fact the most external, or the *muscular zone* of the spiral lamina. Thus the cochlear muscle is broad at its origin from the groove of bone, and slopes above and below to the thin margin in which it terminates, so that its section is triangular, and it presents three surfaces, one towards the groove of bone, and one to each of the scalæ. The surface towards the vestibular scala is much wider than that towards the tympanic scala, and presents, in a band running parallel to and at a short distance from the margin of the membranous zone, a series of arched vertical pillars, with intervening recesses, much resembling the arrangement of the musculi pectinati of the heart. These lead to and terminate in the outer clear belt of the membranous zone, which forms a kind of tendon to the muscle. This entire arrangement is almost sufficient of itself to determine the muscular nature of the structure. If its fibres were of the striped variety no doubt would remain; but its mass, evidently fibrous, is loaded with nuclei, and filled with capillaries, following the direction of the fibres, and in almost all respects it has the closest similarity to the ciliary muscle of the eye." P. 80.

A structure thus remarkable must have, it is evident, some important office in the mechanism of audition. It is thought by Dr. Todd and Mr. Bowman that the action of the muscle must be that of making tense the membranous portion of the lamina spiralis, and so perhaps of adjusting it to the modifications of sound.

" As the ciliary muscle, though of the unstriped variety, adjusts the transparent media of the eye to distinct vision at different distances under the guidance of the will, so it is not impossible that the cochlear muscle may have a voluntary adjusting power, though its precise mode of action as a part of an acoustic apparatus may still remain obscure. On the whole, however, we are more disposed to regard this very interesting structure as having a preservative office, as being placed there to defend the cochlear nerves from undue vibrations of sound, in a way analogous to that in which the iris protects the retina from excessive light. These nerves are acted on principally by vibrations brought through the osseous part of the cochlea, and it is probable that the arrangement of the scalæ is one designed to allow of protective movements of the lamina spiralis by muscular action, under a stimulus reflected from impressions on the auditory nerve." P. 80.

As we have occupied so much space in noticing the various novel and important anatomical facts contained in these Parts, we are compelled, though with reluctance, to pass by the physiological portion of the work. In the last chapter, the authors enter upon the important function of Digestion; but, as the subject is not concluded in the Part now published, it will be preferable to defer any observations we may have to offer in reference to it, till the entire work is completed. In the interim we must, however, urgently recommend those among our readers who are not already in possession of this "Physiological Anatomy," no longer to delay adding it to their library; a recommendation we willingly risk on the preceding extracts, which, limited as they necessarily have been, are still sufficient to indicate that this valuable treatise is quite indispensable to all who aspire to keep up their knowledge with the high standard of the present day.

